# Acute alcohol-induced myocardial inflammation as visualized by cardiac magnetic resonance

**DOI:** 10.1186/1532-429X-13-S1-M4

**Published:** 2011-02-02

**Authors:** Anja Zagrosek, Daniel Messroghli, Sascha Aiche, Olaf Schulz, Steffen Bohl, Ralf Wassmuth, Andre Rudolph, Rainer Dietz, Jeanette Schulz-Menger

**Affiliations:** 1 HELIOS Klinikum Berlin-Buch, Charité Campus-Buch, Humboldt University, Berlin, Germany; 2 Department for Congenital Heart Defects and Pediatric Cardiology, Deutsches Herzzentrum Berlin, Berlin, Germany; 3 Interventionelle Kardiologie Spandau, Berlin, Berlin, Germany; 4 Dept. for Cardiology, Charité-Campus Virchow, Humboldt-University Berlin, Berlin, Germany

## Objectives

We sought to investigate whether binge drinking induces changes of myocardial tissue parameters detectable by cardiac magnetic resonance (CMR).

## Background

Excessive consumption of alcohol (binge drinking) induces a systemic inflammatory reaction including excretion of cytokines. We hypothesised that these immunomodulatory properties of ethanol lead to transient sterile myocardial inflammation detectable by CMR, employing techniques previously established for the diagnosis of inflammatory myocardial injury.

## Methods

31 healthy volunteers participated in the study. In 23 participants binge drinking was simulated by excessive consumption of vodka following a standardized protocol. Participants were examined in a 1.5T CMR-System before, 12 h and 1 week after alcohol intake. Blood alcohol level (BAL) was assessed one hour after alcohol intake. CMR included cine-imaging for cardiac function and volumes (repetition time (TR) 2.9 ms; (TE) echo time 1.2 ms; flip angle 80°; matrix 256×146; 30 phases/R-R-interval, slice thickness/gap 7/3 mm), T2-weighted techniques for detection of cardiac edema (TR 2xRR, TE 65 ms, inversion time (TI) 140 ms, matrix 256x192, slice thickness/gap 15/5 mm), contrast-enhanced T1-weighted global relative enhancement (gRE, TR 1137 ms, TE 14 ms, slice thickness 8 mm), and late gadolinium enhancement (LGE) for detection of myocardial scarring (TR 5.5 ms, TE 1.4 ms, TI 225-320 ms, matrix 256x192, slice thickness/gap 7/3 mm). Eight control participants underwent CMR twice within twenty-four hours to test for effects of repeated contrast exposure. High-sensitive cardiac Troponin I (cTnI) was evaluated in a subgroup of 12 participants.

## Results

The mean BAL after drinking was 1.1±0.3‰. In CMR, a significant increase in myocardial signal intensity on T2-weighted images (1.9±0.2 vs. 1.7±0.2, p=0.001) and on gRE-images (6.7±2.9 vs. 4.2±1.5, p=0.008) was observed the day after drinking. Both abnormal CMR parameters returned to baseline within one week. No areas of focal LGE-enhancement were detectable, cardiac function and volumes remained unchanged. No gender related differences in the course of CMR parameters were found. The control group showed no changes in CMR parameters in two consecutive examinations. cTnI increased in 6 of 12 (50%) participants after binge drinking (0.04±0.03 µg/l vs. 0.01±0.00 µg/l, p=0.03).

## Conclusion

This study suggests that even a single episode of binge drinking leads to a transient myocarditis-like inflammation of the myocardium, which is detectable by CMR.

**Figure 1:**
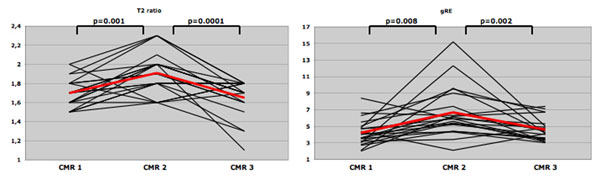
Individual course of T2 ratio (left) and gRE (right) before (CMR 1), twelve hours (CMR 2) and one week (CMR 3) after binge drinking.

